# Globe Artichoke (*Cynara scolymus* L.) By-Products in Food Applications: Functional and Biological Properties

**DOI:** 10.3390/foods13101427

**Published:** 2024-05-07

**Authors:** Raffaella Colombo, Giulia Moretto, Vanessa Pellicorio, Adele Papetti

**Affiliations:** 1 Department of Drug Sciences, University of Pavia, Viale Taramelli 12, 27100 Pavia, Italy; raffaella.colombo@unipv.it (R.C.); giulia.moretto@universitadipavia.it (G.M.); vanessa.pellicorio@unipv.it (V.P.); 2 C.S.G.I., University of Pavia, Viale Taramelli 12, 27100 Pavia, Italy

**Keywords:** circular economy, artichoke by-products, valorization, bioactives, functional properties, food additives, functional foods, food supplements, animal feed

## Abstract

Globe artichoke (*Cynara cardunculus* var. *scolymus* L.) is widely cultivated in the Mediterranean area and Italy is one of the largest producers. A great issue is represented by its high amount of by-product, mainly consisting of external bracts and stems, but also of residual leaves, stalks, roots, and seeds. Artichoke by-products are rich in nutrients (carbohydrates and proteins) and bioactive compounds (polyphenols and terpenes) and represent potential ingredients for foodstuffs, functional foods, and food supplements, due to their functional and biological properties. In fact, artichoke by-products’ components exhibit many beneficial effects, such as dyspeptic, prebiotic, antioxidant, anti-inflammatory, antiglycative, antimicrobial, anticarcinogenic, and hypolipidemic properties. Therefore, they can be considered potential food ingredients useful in reducing the risk of developing metabolic and age-related disorders. This work summarizes the economic and environmental impact of the recovery and valorization of artichoke by-products, focusing on rheological, physical, and biological properties of the different components present in each by-product and their different food applications.

## 1. Introduction

Globe artichoke (*Cynara cardunculus* var. *scolymus* L.) belongs to the *Asteraceae* family and is the oldest cultivated artichoke variety in the Mediterranean area, representing a lead food component of the diet. Its global production amounts about to 1500 Kt and the edible parts consist only of heads (flowers) and, partially, stems, generating a large amount of by-product (80–85% of fresh weight material), mainly composed of external bracts and stems, but also residual leaves, stalks, roots, and, to a lesser extent, seeds. Italy, Spain, and France are the main globe artichoke producers; Italy is currently the leader not only in the production of globe artichoke (390 Kt, corresponding to 26% of the world production), but also in the recovery and valorization of its by-products [1,2,3]. FAO estimated that, annually, 1.3 Bmt of edible food is lost or wasted, with a production of around 190,000 Kt of by-products (leaves, seeds, brans, pomaces, meals, cakes, and raw materials) and, accordingly, a great impact on worldwide cost ($750 B/year) [1,4]. In fact, nowadays, the environmental and economic impact of food-waste disposal represents an ever-growing issue and, for this reason, the United Nations is continuing to encourage the recovery policies and circular-economy plans for waste reduction and re-use, to guarantee food security and protect human health and the environment by decreasing air, water, and soil pollution [4,5,6,7,8]. The actual trend of circular economy is based on the production of bioenergy, biocomposite material, or bioactive compounds, and the search for efficient and environmentally friendly methodologies with sustainable procedures and competitive costs in comparison to those incurred for food-waste disposal [2,6].

*C. cardunculus* L. is widely cultivated for its large immature inflorescences (30–40% of fresh weight material), also named capitula or heads, which represent the edible parts together with small stems and fleshy bracts. They are mainly composed of fiber (inulin and pectin), minerals (K, P, Ca, Mg, Na, Zn, Fe, Cr, and Mn), vitamins (E, C, B3, B6, B8, and B9), polyphenols such as hydroxycinnamic acid derivatives (monocaffeoylquinic, CQA and dicaffeoylquinic, and diCQA), flavones (apigenin, luteolin, and their glycosides), and anthocyanins (cyanidin 3,5-*O*-diglucoside, cyanidin 3-*O*-glucoside, cyanidin 3,5-*O*-malonyldiglucoside, cyanidin 3-*O*-(3″-*O*-malonyl)glucoside, and cyanidin 3-*O*-(6″-*O*-malonyl)glucoside), terpenoids, saturated and unsaturated fatty acids, aspartic endopeptidases (cardosins and cynarases), and peroxidases.

Globe artichoke-cultivation generates about 33 t/Ha of waste, and only a low percentage (15–30%) of artichoke by-products (ABPs) can be valorized (bracts and stems), while 70–85% is composed of residual biomass from cultivation [3,9,10]. This biomass consists of residual leaves, stalks, and roots and mainly contains carbon, nitrogen, ash, proteins, cellulose, inulin, and lignin [9].

ABPs are especially rich in carbohydrates (cellulose, hemicellulose, and inulin) and derivatives (lignin), but also in sterols (stigmasterol and β-sitosterol), polyphenols (mainly CQAs and diCQAs), terpenoids (mono-, sesqui- and triterpenes), vitamins (E and C), and, to a lesser extent, carotenoids. Over the years, ABPs were mainly studied as source of fibers and antioxidants [3,11,12,13], which are well known for their involvement in the risk reduction of different diseases related to lipid and liver metabolism, cancer, and age-related disorders [10,14,15].

Bracts and stems are rich in hydroxycinnamic acid derivatives and flavones such as luteolin and apigenin, which are the main compounds responsible for ABPs health effects, i.e., its antioxidant, anti-inflammatory, antimicrobial, and anticancer activities [10,11,16]. The polyphenol content is strictly related to the extraction process, which can also affect the bioactivity, mainly the antioxidant power [17]. In addition, ABPs are good source of proteins, such as aspartic protease enzymes with coagulant properties and peroxidases possessing antioxidant activity [18,19,20]. Bract and stem insoluble (mainly cellulose, hemicellulose, and lignin) and soluble (mainly inulin, pectin, gums, and β-glucans) fiber content is about 35 g/100 g dry matter (dm) and 20 g/100 g dm, respectively. In particular, inulin is responsible for ABPs prebiotic activity, and its content is strictly dependent on cultivar and location [16,21].

The main benefit deriving from the re-use of such a low amount of valorizable ABPs (bracts and stems) is represented by the simple and cheap processing needed to treat them to obtain the bioactives useful for different food or non-food applications. In fact, in the past, ABPs were only used as animal feed and fiber manufacturing, but recently, they have been considered ingredients with high nutritional value and health-promoting effects or functional properties and, therefore, potentially useful to produce foodstuffs, functional foods, and food supplements [11,15,22,23,24].

In the economic re-cycle of by-products, all the procedures of their preparation must be selected and adopted with the aim to maximize yield, preservation, quality, and bioactivities. In fact, there is a great need to valorize agricultural by-products into food products as they represent a very cheap source of different compounds. Therefore, it is mandatory to adopt efficient recovery methodologies, suitable for re-use policies, and with competitive cost in comparison to that displayed by high-cost waste-disposal treatment [24,25,26,27,28]. In particular, the quality of the starting raw material and the selection of the extraction method are fundamental. A good solution to obtain stable and high-quality by-products is represented by the silage fermentation process, in which the increase of lactic acid bacteria (mainly *Lactobacillus acidophilus* and *Bifidobacterium bifidum*) can affect the content or release of nutrients and bioactives [19,26,29,30]. Over the years, to develop a real global circular-economy model able to minimize the environmental and economic impact, the application of green extraction methodologies represented a valid strategy. This allowed for overcoming the limitations of traditional technologies and reducing the reagent cost deriving from the by-products’ re-use, also being time saving [31,32]. Regarding ABPs, the use of green solvents (water, methanol, ethanol, and natural deep eutectic solvents-NADES) [33] and the applications of heat treatment [13,32,34], microwaves (microwave-assisted extractions-MAE) [35], pressure (pressurized-liquid extraction-PLE or pressurized-hot water extraction-PHWE) [36,37,38], or ultrasounds (ultrasound-assisted extraction-UAE) [39,40,41], often combined, were explored, obtaining more rapid, efficient, and selective extraction processes potentially scalable at industrial level [24,32].

**Figure 1:**
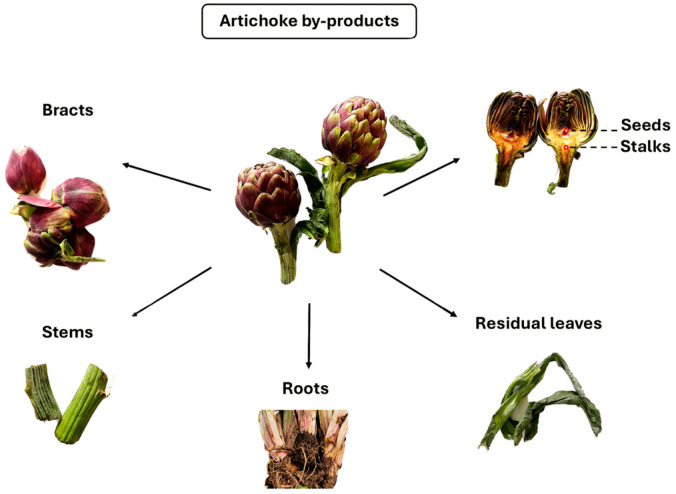
Different by-products from globe artichoke.

In the first part of this review, we addressed all ABPs (Figure 1), discussing their specific chemical composition and functional and/or biological properties; differently, in the second part, we discussed the different food applications of each ABP extract as a food additive and ingredient for functional foods, food supplements, and animal feed. We also briefly commented on the main steps of ABPs’ preparation, which can be adopted to optimize each procedure and obtain efficient recovery. ABPs represent a promising and versatile source of compounds useful in food and non-food applications.

## 2. Chemical Composition

### 2.1. Globe Artichoke Main By-Products: Bracts and Stems

Bract content of bioactive compounds (particularly polyphenols) and nutrients (such as proteins and inulin) is strongly related to the bract position, as demonstrated by Ruiz-Cano (2014) for the inner bracts near to the artichoke heart, which are the richest in bioactives (phenolic acids, flavonoids, caffeoyl derivatives, and flavones), proteins, and inulin [42]. In addition, the concentration of polyphenols, mainly CQAs and diCQAs (1–8%, *w*/*w* dm) and flavones (luteolin and apigenin), is strictly dependent on tissue physiological stage, with the highest amount detected in the young tissue. Clones and landraces also affect their concentration, reaching total phenolic content (TPC) values ranging from 8.5 to 12.7 g/kg dm, with only about 10% represented by flavones [11]. 5-CQA, 1,5-diCQA, luteolin-7-*O*-rutinoside, and luteolin-7-*O*-glucoside are the main detected compounds. Stems are particularly rich in CQAs, differently from bracts, which are richest in flavones (mainly luteolin) [11,14,16,43]. The polyphenolic extract concentration is affected by different parameters; generally, but more markedly for the inner bracts, it is positively influenced by pre-heating treatment, probably because of an inactivation of oxidative enzymes (oxidases and peroxidases), thus preserving these compounds from oxidation [42]. Temperature and time are other key factors, probably due to an increase of the compounds’ water diffusivity and solubility, as demonstrated for 5-CQA recovery, which was optimized by extracting at 60–75 °C for 60 min [32,44]. In addition, the TPC content may differ in relation to the organic modifiers used in the extraction procedure; in fact, the highest concentration of polyphenols and the best antioxidant activity can be obtained using 50–75% ethanol solution [38,45]. Differently, higher ethanol percentages (96%) can decrease TPC content and a careful set-up of temperature (40 °C for bracts and 120 °C for stems) is needed to increase the diffusivity and the mass transfer and, therefore, to maximize the extracted TPC [13]. Generally, medium-high temperatures (≤120 °C) are suggested when using PLE with ethanol (>85%) [36]. Temperature becomes even more important when using PHWE, as well as the modifiers’ percentage, to obtain an efficient recovery of 5-CQA, 1,5-diCQA, and flavone (luteolin and apigenin) glycosides. In fact, for example, a low ethanol percentage (≤10%) and temperature (20 °C) allow for obtaining high extraction yield of 5-CQA from artichoke bracts. In PHWE, the numbers of cycles and the extraction time can also affect the extraction process [38]. When UAE is adopted, an optimization in the use of ultrasounds is required, as highlighted for the recovery of bract polyphenols (power: 240 W and time: 60 min) [40,41,46]. Stems contain also condensed tannins which can be extracted with different yields according to the polarity of the solvent used, generally obtaining about 309 μg catechin equivalent (CE)/g extract (dm) using methanol and 540 µg CE/g extract (dm) using butanol [12].

As previously mentioned, bracts and stems are rich in complex carbohydrates, represented both by insoluble fiber such as cellulose, hemicellulose, and lignin (35 g/100 g dm) and soluble fiber such as inulin, pectin, gums, and β-glucans (20 g/100 g dm) [47,48], and proteins (10–18%), whose content decreases when heating treatments are applied during the extraction procedure [18,42]. Bracts are mainly composed of cellulose (38.5%, *w*/*w* dm), hemicellulose (23.8%, *w*/*w* dm), lignin (6.6%, *w*/*w* dm), and inulin, which is also present in high amounts in stems and whose concentration is related to cultivars (20–30%, *w*/*w* dm) [16,34,42,49]. To increase inulin recovery, a pre-heating treatment (70 °C, 5 min) before the extraction can be performed with stronger effect on stems (53%, *w*/*w* dm) than on bracts (44.7%, *w*/*w* dm) [34,50]. In addition, inulin degree of polymerization (DP) is strictly dependent on the harvest season and storage conditions, and, therefore, its prebiotic properties can be highly variable. For example, the inulin extract is more stable (for at least few days) when stored at 3–7 °C than at room temperature [51]. Intestinal bacteria (*Lactobacillus plantarum* and *B. bifidum*) fermentation and prebiotic effects are strictly related to the high inulin content and are accelerated in the presence of pectins with a low degree of methyl-esterification (DM) [50].

Bracts also contains sesquiterpene lactones, mainly cynaropicrin (20.8 ± 0.5, mg/g dm), grosheimin, 11β,13-dihydrocynaropicrin, deacylcynaropicrin, and isoamberboin, which were extracted using ethyl acetate with very low extraction yields (0.00029–0.066%). These compounds have important effects on gastrointestinal digestion and motility, and cynaropicrin, especially, is also responsible for the typical bitter taste of artichoke [52,53]. Differently, in stems, only a low amount of sesquiterpene lactones, mainly cynaropicrin (2.7 ± 0.2, mg/g dm), is present [53].

Regarding proteins, stems are extremely rich in peroxidases (7.21%), which are enzymes catalyzing the oxidation reactions in the presence of hydrogen peroxide and are involved in many metabolic processes [20].

### 2.2. Other Globe Artichoke By-Products: Residual Leaves, Stalks, Roots, and Seeds

Artichoke residual leaves are rich in CQAs, diCQAs, and flavonoids, which were successfully extracted using PHWE with an extraction yield similar to that obtained for bracts, after temperature, ethanol percentage, time, and number of cycles optimization [38]. Residual leaves also contain aspartic protease enzymes (cardosins and cyprosins) useful as rennet in many food products for their coagulant properties [19,54,55].

Stalks contain cellulose, hemicellulose, and lignin, and their content differs depending on the dimensions; in fact, thin stalks contain a higher percentage of hemicellulose (24.1% dm), while the thick ones are the richest in cellulose (49.3% dm) and lignin (13.2% dm) [49].

A high amount of inulin is generally present in roots, but its concentration is strictly related to cultivar and location; thus, it ranges from 6.5 to 20.9 g/100 g, and it can be optimized using UAE, which allows for obtaining an efficient and rapid extraction due to an increased cavitation and mass-transfer process [56,57,58,59,60].

**Figure 2:**
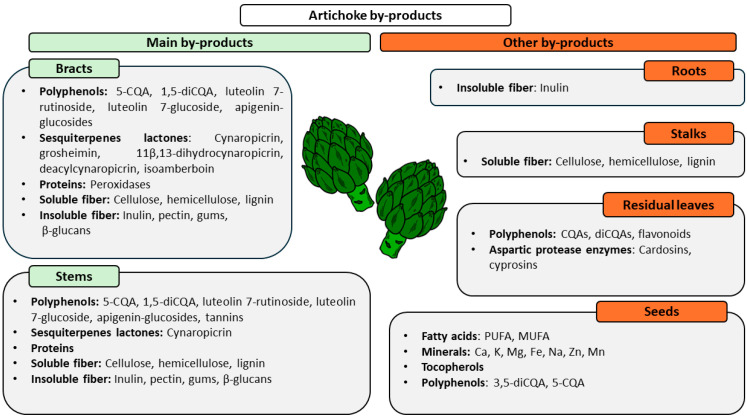
Chemical composition of each specific by-product.

Finally, artichoke seeds represent the less abundant ABPs. Depending on the different genotypes, protein and fat content ranges from 25.7 to 30.4 g/100 g dm and from 17.3 to 23.7 g/100 g dm, respectively. Fatty acids are mainly represented by polyunsaturated (PUFA) (60.43–70.64%) and monounsaturated fatty acids (MUFA, 13.08–19.71%). As regards minerals, Ca (734–1583 mg/100 g dm), K (493–880 mg/100 g dm), Mg (241–809 mg/100 g dm), and Fe (9.2–16 mg/100 g dm), and, in lower concentrations, Na (12–24 mg mg/100 g dm), Zn, and Mn are present. Tocopherols are also present, but in even lower amounts (1.83–4.46 mg/100 g dm). Among polyphenols, 5-CQA (22.5–35.8 mg/100 g dm) and 3,5-O-diCQA (197–418 mg/100 g dm) are the main ones represented [61]. Figure 2 summarizes ABPs’ chemical composition.

## 3. Artichoke By-Product Processing

The natural and industrial methods of processing by-products differ widely and affect by-products’ chemical composition and, accordingly, their biological and functional properties. This section mainly focused on ABPs’ storage, thermal treatment, and bioactives extraction, briefly discussing the most efficient procedures reported in the literature to obtain the highest nutrient and bioactive yields in a short time and at a low cost.

As regards storage, the silage fermentation process is a well-known procedure to preserve and stabilize food products and agro-food by-products for the long term. It is based on the natural lactic fermentation in anaerobic conditions, after temperature and pH optimization, and the type of fermentative containers selected [22,25]. In ensiled ABPs, which are rich in inulin and fructooligosaccharides (FOS), it is evident that there is an inhibition of harmful bacteria and a better growth of lactic acid bacteria (*Lactobacillus*, *Lactococcus*, *Serratia*, and *Weissella*). Therefore, this fermentation process improves the release of short chain fatty acids (SCFAs), mainly acetic, propionic, and butyric acids, possessing antimicrobial, antioxidant, anti-inflammatory, and antiproliferative properties [26,29]. ABPs’ ensilage process in commercial round bale silos has been indicated as a suitable practice to ensure prolonged shelf life by reducing enterobacteria, clostridia, and yeast contamination and the loss of nutrients and bioactives (such as proteins and polyphenols), as well as improving the organoleptic properties (smell and palatability). The result is the production of high quality, stable, and safe products [29].

Blanching and boiling can be applied to improve the nutrients or bioactives extraction, as reported by Ruiz-Cano et al. [42]. In fact, both pre-treatments increased inulin and polyphenols extraction yields, but with higher yields when boiling was applied than when blanching was. In addition, different cutting sizes of bracts affected the polyphenols extraction yields whether blanching or boiling was used. Differently, the fat content did not significantly change when either of the two pre-treatments was applied.

As above mentioned, the use of green technologies to extract bioactive compounds from ABPs agrees with the fundamental issues of environmental and economic policies. Nowadays, pressurized liquids represent one of the most promising extraction methodologies, characterized by a high reproducibility and reduced extraction time and solvent consumption, which are important factors to be considered in scaling-up the process [38]. PLE is based on the use of eco-friendly and food-grade solvents (water or hydroalcholoc mixtures) at high pressures (around 100 atm) and temperatures, maintaining the solvent in a liquid state, but reducing solvent viscosity, thus modifying penetration and mass-transfer capacity and rate. This increases extraction yield and selectivity, mainly in regard to polar compounds such as phenolic acids but can cause a degradation of molecules sensitive to oxidation. To avoid this, a suitable treatment to reduce or eliminate oxygen must be considered (for example, sonication could be an option). The high-speed process and the low solvent volume lead to a cost reduction, thus offsetting the potential instrument cost, which can be more expensive especially if the technology is able to reach high pressure values [36,37,38].

## 4. Biological Properties

ABPs’ components were widely studied for their different activities, mainly antioxidant, anti-inflammatory, prebiotic, antimicrobial, and antiproliferative, with potential applications in many important chronic diseases. To investigate the bioactivity, it is important to consider how the potential active compounds can be bioaccessible following the digestion process. Therefore, in vitro studies on bioaccessibility and bioavailability are generally performed to obtain more predictive behaviors of active molecules in vivo; for this purpose, static or dynamic systems are applied to mimic the specific gastrointestinal environments [62,63,64,65,66,67,68,69].

### 4.1. Hypolipidemic Activity

ABPs were also studied for their potential effects on hyperlipidemia, a risk factor of important chronic diseases, from cardiovascular to metabolic disorders [44,70]. The action on lipid metabolism is essentially due to the presence of fiber components (inulin, arabinans, arabinogalactans, and xyloglucans), as they possess swelling properties which could reduce the lipid absorption at intestinal level. In fact, supplementation with 20% ABP fiber decreases triglycerides (TGs) and total cholesterol, directly affecting its synthesis, and reduces LDL and liver lipid accumulation, showing a positive effect for Syrian hamsters fed a high-fat diet and suffering for hepatic steatosis [71]. Furthermore, the hypolipidemic activity was also associated with bract sesquiterpene derivatives (cynaropicrin, aguerin B, and grosheimin) that reduce TGs levels in a specific animal model (lipid-loaded mice), with a direct action on gastric emptying and absorption [72].

Another interesting study is related to the capacity of artichoke bract polyphenols to reduce LDL’s oxidation products, such as malonyldialdehyde, triglycerides, and cholesterol, in alloxan-induced diabetic mice [73].

### 4.2. Hypoglycemic and Antiglycative Activity

As regards the hypoglycemic effects, 5-CQA is the main polyphenol involved in the direct inhibition of glucose-6-phosphate translocase (isolated from liver microsomes of male rats), a key enzyme in the gluconeogenesis, which results in the reduction of glucose intestinal absorption [74].

The antiglycative activity concerns the inhibition of the glycation process, which is involved in the development of complex pathways generating different molecules, such as dicarbonyl compounds, that are toxic mainly for heart, liver, and kidney, as well as advanced glycation end products (AGEs) that are associated with oxidative processes and correlated to many age-related disorders from diabetes to neurodegenerative diseases. Aqueous, alcoholic, and hydroalcoholic outer-bract and stem extracts were investigated for their potential use as antiglycative agents by different in vitro assays specifically set up to reproduce the stages of the glycation process. Different polyphenols were extracted depending on the ethanol percentage used and, therefore, the different extracts had different activity. In fact, the ethanolic extract was rich in 5-CQA, caffeic acid, and 1,5-diCQA, while the aqueous extract mainly contained 1-CQA, 3-CQA, and 1,3-diCQA. Both extracts inhibited the initial stage of the reaction, but the ethanolic extract had higher activity when ribose was used in the model system to generate AGEs, differently from the aqueous extract, which had the best activity in the presence of glucose and fructose [44].

It is important to underline that CQAs activity is strongly reduced after the digestive process; in fact, 5-CQA, which is the most promising antiglycative agent, loses its effect, as evident from the bioaccessibility results obtained by gastrointestinal-process simulation performed using both static and dynamic digestion approaches [44,75].

### 4.3. Anti-Inflammatory and Antioxidant Activities

Today, ABPs’ anti-inflammatory activity is well known, and it is very important for the prevention of chronic debilitating pathologies. The inflammation occurs as a defense mechanism in the case of acute injury or infection, involving complex pathways and many pro-inflammatory mediators (cytokines, nitric oxide-NO, histamine, prostaglandins, and leukotrienes); however, like oxidative stress, it can be even more dangerous when it becomes a chronic condition, as it is a risk factor for several critical diseases, such as arthritis, cardiovascular, and neurodegenerative disorders [76]. Also, in these conditions, polyphenols are among the lead compounds tested for their capacity to prevent or delay the formation of pro-inflammatory mediators. For example, good results were registered for an aqueous stem extract obtained by applying the pulsed electric field (PEF) treatment (used to increase the cell membrane permeability and disruption). This extract, mainly rich in 5-CQA (54%), reduces the effect on the expression and secretion of Interleukin-6 (IL)-6 and monocyte chemoattractant protein (MCP)-1/C-C motif chemokine ligand 2 (CCL2) in human THP-1 macrophages, when used as an inflammatory cell model [77]. A strong reduction in prostaglandins and other inflammatory mediators (C-reactive protein-CRP) and fibrinogen), and in the migration of inflammatory cells, was registered for a bract ethanolic extract. In this case, a carrageenan-induced paw edema was used as a model to induce the acute inflammatory response, being suitable for evaluating the expression of cyclo-oxygenase (COX)-2 and prostaglandins, as well as vascular permeability [45].

The anti-inflammatory properties were also attributed to sesquiterpene lactones extracted from leaves because of their inhibition of NO production in macrophage-like RAW264.7 cells with an effect on inducible nitric oxide synthase (iNOS). Fundamental for this action is the presence of the α-methylene-γ-butyrolactone chain and acyl portion with an α, β-unsaturated carbonyl group at Position 8 in the sesquiterpenes skeleton [52].

Finally, pectins isolated from bracts, stems, and leaves exhibit anti-inflammatory activity in mice with colitis induced by dextran sulfate sodium; the parameters affecting the action are the presence of arabinose and, mainly, of galactose as well as the degree of esterification and the molecular weight (MW). The supposed mechanism of action is the reduction in cytokines and iNOS in addition to changes in the transmembrane receptors involved in immune responses [78].

Inflammation is strictly linked to oxidative damage caused by free radicals, Reactive Oxygen Species (ROS), and Reactive Nitrogen Species (RNS), and many plant extracts rich in polyphenols were investigated for their potential antioxidant activity, including ABPs [79,80]. The protective activity of bract and residual leaf extracts on liver damage caused by oxidative processes was tested on an hepatocarcinoma cellular model (human HepG2 cells) and was mainly attributed to 5-CQA, 1,3-diCQA, 1,5-diCQA, and luteolin. The results highlighted a direct correlation between the polyphenolic content and the antioxidant effect, with the strongest antioxidant action registered for 1,3-diCQA [16]. Similar results were obtained testing a 60% *v*/*v* methanolic extract of bracts and stems. In fact, the high 5-CQA concentration is related to the high antioxidant activity measured with different in vitro test systems (2,2-Diphenyl-1-picrylhydrazyl (DPPH) assay, 2,2′-azino-bis(3-ethylbenzothiazoline-6-sulfonic acid (ABTS) radical assay, and ferric reducing antioxidant power (FRAP) assay). In addition, 5-CQA and 1,3-diCQA have significantly reduced ROS production when tested using Caco-2 cells as a model to evaluate the antioxidant capacity against hydrogen peroxide damage on intestinal cells [81].

The same antioxidant in vitro assays were used to test aqueous extracts of bracts and stems rich in 5-CQA and 1,5-diCQA with good results. Similar results were also registered on plant cells (tobacco BY-2 cells) used as a model of epithelial tissue [41].

### 4.4. Anti-Proliferative Activity

Few studies tested ABPs’ anti-proliferative properties. Recently, bract and stem hydroalcoholic extracts were tested at different concentrations (0.5–2000 mg/mL) on MCF-7 breast cancer cells and Caco-2 colorectal carcinoma epithelial cells. Interesting results were obtained. In fact, at low concentrations, cell viability increases, probably due to the presence of other compounds, such as saccharides that promote cell growth. Conversely, MCF-7 and Caco-2 cells’ exposure to higher concentrations (750 mg/mL and 1500 mg/mL) results in a reduced cell viability (26.2% and 47%, and 19.3% and 30.4% for MCF-7 and Caco-2, respectively) [82]. Caco-2 cells were also used as a model to test a bract ethanolic extract, obtaining a higher effect (IC50 143 μg/mL) than that registered for human colon carcinoma (HCT116) (IC50 234 μg/mL) and mammalian cells (Vero) from African green monkey kidney cell lines (IC50 329 μg/mL) [83]. These results confirm the previous ones obtained by Salama et al. (2013), who evaluated the cytotoxic properties of bract methanolic extract on the HepG2 cell line, obtaining a strong effect (IC50 69 μg/mL) [84]. The action mechanism is probably the regulation of apoptosis-related proteins, or the deregulation of the cell cycle, operated by the phenolic compounds present in the extract, as also reported by Mosehly et al. (2023) [83].

### 4.5. Antimicrobial Activity

Several authors investigated ABPs’ antimicrobial properties and, nowadays, the search for plant-derived natural products is becoming widespread to improve food quality and safety as a consequence of frequent food microbial contamination. In addition, the increase in antibiotic resistance represents a big issue for public health [85,86].

Artichoke floral stem extracts obtained using different solvents, i.e., methanol, ethanol, butanol, and ethyl acetate, were tested against three Gram-negative (*Enterococcus faecium*, *Salmonella typhimurium*, and *Escherichia coli*) and two Gram-positive (*Staphylococcus aureus* and *Streptococcus agalactiae*) bacterial strains, and *Candida albicans* yeast. All the extracts possessed antimicrobial properties, with the exception of *S. typhimurium*, *E. coli*, and *C. albicans*, and the activity was mainly attributed to CQAs and flavones. In particular, the methanolic extract was the most active, especially toward *S. aureus* and *E. faecum* strains (inhibition zone of 30–31 mm), showing the lowest minimum inhibitory concentration, MIC (1 mg/mL) and the highest bactericidal concentration (1.5–2 mg/mL) [12].

In addition, bracts have antimicrobial activity, as evident from the results obtained for an ethanolic extract rich in polyphenols that is able to inhibit the growth of some foodborne pathogenic bacteria (*Salmonella enterica*, *Salmonella typhi*, *E. coli*, *S. aureus*, *Staphylococcus sciuri*, *Bacillus cereus*, and *Pseudomonas aeruginosa*), although the highest activity was observed against *S. enterica* and *S. typhi* (inhibition zone of 11.3 and 9.5 mm, respectively) with a MIC value of 0.08 mg/mL and 0.27 mg/mL, respectively. This extract is also active against fungi, mainly *Aspergillus ochraceus*, *Aspergillus niger*, and *Fusarium proliferatum* (inhibition zone of 8.0 mm) with MIC values of 1.17, 2.33, and 1.5 mg/mL, respectively [14].

### 4.6. Prebiotic Activity

The role of fiber is fundamental for the balance of intestinal flora and the control of lipid metabolism and cholesterolemia. The inulin-type fructans are widely studied as prebiotics for their stimulating effect on the growth of *L. acidophilus* and *B. bifidum* in the colon, which promotes the formation of SCFAs and the absorption of minerals (calcium and magnesium) [87].

Bracts and stems are good source of inulin (70%) with high DP (32–42), which has a greater stimulation action on the growth of different Bifidobacteria and Lactobacilli than the commercial FOS. In particular, an increase in OD_600_ (from 0.1 to 0.5–1.5, depending on the strain) was observed after 48 h of incubation at 37 °C under anaerobic conditions, indicating a proliferation of Bifidobacteria and Lactobacilli [21]. Pectin extracted from the same by-products was also investigated for its potential effect on the growth of Bifidobacteria, Lactobacilli, and Bacteroides/Prevotella. An enzymatically modified pectin characterized by low MW, higher galactose (Gal) and arabinose content, and a higher Gal:Rhamnose (Rha) ratio was more active on the cited bacteria growth than common pectin (an increase of 9.82, 9.17, 9.45 log10 bacteria/mL was observed for modified pectin vs. 9.50, 8.92, 9.23 log10 bacteria/mL for common pectins, for the three microbial populations mentioned above, respectively), even if both identically promote the formation of SCFAs (acetate, propionate, and butyrate), thus highlighting the fact that this action is not related to pectin structural properties [88]. The use of acid or enzymatic hydrolysis to extract inulin and pectin was compared in another study. The results indicated that higher content of inulin and pectin can be obtained using enzymes, thus reaching a great effect in promoting the growth of *Lactobacillus plantarum* 8114 and *Bifidobacterium bifidum* 11863, with a cell density of 2.94 ± 0.19 and 1.90 ± 0.09 log10 CFU/mL, respectively [50].

Therefore, the dietary fibers in ABPs (mainly inulin and pectin) could be considered promising efficient prebiotics once well isolated and purified, as the presence of other secondary metabolites with antimicrobial properties may interfere with the bifidogenic effect of these fibers [26].

All the biological properties of different ABPs (bracts, stems, and residual leaves) are reported in Table 1.

## 5. Functional Properties

Pectin and inulin extracted from bracts and stems can be also used as additives in food and non-food formulations, influencing the brightness (L*) and slight color of the final products. Bract and stem extracts exhibit a common yellowish (b*: blue to yellow) and reddish (a*: green to red) tone, with a high intensity for stems, which appear darker than bracts. The extraction can affect the color, too; in fact, by comparing extracts obtained by acid and enzymatic hydrolysis, differences in brightness, color, and tones (red, blue, green, and yellow) can be registered, and the use of enzymes results in an increase in brightness and color, without tones changing. The presence of chlorophyll can modify the ABPs’ color properties, decreasing brightness and red/green tones [89,90].

ABPs can be also exploited in the food industry as rheological modifiers due to their high content in soluble fibers, i.e., inulin and pectin. Inulin is a fructan-type polysaccharide composed of (2→1) linked β-D-fructosyl residues with the end part usually represented by an (1↔2) α-D-glucose. Its DP and MW affect the rheological properties and potential applications as a gelling or emulsion agent [89,91]. Pectin is a polysaccharide backbone of α-1,4-linked D-galacturonic acids (GalA), made of three polysaccharide domains: homogalacturonic (HG), rhamnogalacturonic-I (RG-I), and rhamnogalacturonic-II (RG-II). The DM of GalA, the presence of calcium or cosolutes (i.e., sucrose and acidifiers), or the distribution of unesterified GalA residues can change pectin gelling properties [34]. Another important factor that affects the rheological properties (gelling, thickening, or stabilizing agent) is represented by the extraction conditions. In fact, a thermal treatment before the extraction process or enzymatic hydrolysis can increase fiber recovery from stems. This fiber is mainly characterized by 70% inulin, with a low amount of low-DM (15–33) pectin; it exhibits high or low viscosity, depending on whether a high or low shear rate is applied, as pseudoplastic material. The high amount of inulin, together with the presence of low-DM pectin and calcium (40 mg/g pectin), contribute to form rigid gels. Conversely, the unheated extracts give origin to different DM pectins, forming creamy gels with calcium [34]. In fact, the degree of viscosity is strictly dependent on the extract preparation method. In addition, different procedures of hydrolysis can result in various inulin DP and pectin DM with, consequently, different properties. For example, fiber obtained from bracts and stems using either acid hydrolysis or acid hydrolysis in combination with enzymatic procedures (protease and cellulase) is characterized by a high content of low-DM pectin and of inulin with different DP, with a high capacity for interacting with calcium, giving origin to strong and well-structured gels with different calcium crosslinking degrees. An important difference in the fiber obtained using the two hydrolysis approaches consists in the viscosity value, as the action of enzymes on cell walls causes the release of polysaccharides with lower thickening capacity and viscosity in comparison to those obtained with the acid hydrolysis alone [23]. Inulin extracted from artichoke roots is characterized by a high-DP value (32), similar to that of commercial inulin obtained from chicory roots, and forms gels with similar properties, resulting in structures with a homogeneous network. Also, in this case, the extraction process influences the fiber gel hardness, with lower values for inulin obtained by applying ultrasounds than for those obtained using hot water. In fact, ultrasounds act by releasing interferents that compromise the formation of inter-chain interactions [58].

Low concentrations of artichoke bract extracts (2%) exhibit emulsifying properties and increase the emulsion stability, reducing coalescence and flocculation processes. This effect is always attributable to the presence of soluble fiber (inulin and pectins). However, a positive effect in terms of coalescence reduction is also proved by ABPs’ (mainly bract) amphiphilic proteins and insoluble fiber, thus contributing to stabilize the oil–water (O/W) interface. In fact, bracts’ properties were also investigated for their potential use as additives to stabilize lipids in O/W emulsion and to reduce lipid oxidative processes, as, for example, in PUFA microcapsules’ preparation [92]. In addition, it was demonstrated that the functional properties of stem and bract extracts depend on the presence of chlorophyll. In fact, its removal is fundamental to obtain products with an ideal value of low water activity (a_w_ 0.6) and high water (WHC)- and oil (OHC)-holding capacity. These characteristics not only contribute to reducing the microbial growth and to increasing food products’ shelf life, but also to improving water retention during baking processes, also influencing the product palatability [89].

Nowadays, the addition of natural antioxidants as preservatives instead of synthetic compounds is widely investigated to satisfy the demand of consumers, who require safer products [93]. In this context, ABPs represent the ideal preservatives of food products to stabilize and increase shelf-life, thanks to their antioxidant properties. For example, artichokes bracts, spikes, and receptacles were used to reduce the oxidation of canola oil by at least 15%, 24%, and 72%, respectively, thus showing a higher activity than that of synthetic preservatives as butylated hydroxytoluene (BHT) [94]. Similar results were also obtained by adding an ABP extract to tomato juice, obtaining a promising antioxidant effect and improved shelf life [95]. In Table 2, all the functional properties of ABPs (bract, stem, and root) are summarized.

## 6. Food Additives

ABPs respond to the request of new natural food additives and, in the last decades, they have been widely used in dairy product production (yogurt, milk, and cheese) mainly for their rheological properties, but also for their prebiotic activity, thus ensuring the survival and activity of probiotic bacteria during fermentation and storage. In particular, the addition of artichoke leaf extracts rich in inulin can change the rheological properties because inulin acts as thickener, reducing the serum separation and yogurt syneresis and promoting the formation of complexes among yogurt protein aggregates which can be stabilized by hydrogen bonds [96]. Further, recent studies have shown that inulin addition to stirred bio-yogurt improves its viscosity and creaminess. The increase in viscosity is probably due to the effect of inulin in the formation of casein micelle bonds with particular number, strength, structure, and spatial distribution. This makes inulin a promising fat replacement [60]. However, the addition of leaf extract negatively affects the physical properties of the food product that appears yellow colored and with bitter flavor, probably due to presence of sesquiterpene lactones. Certainly, this extract promoted *L. acidophilus* LA-5 and *B. lactis* BB-12 growth during fermentation and storage, thanks to its components (i.e., proteins, free amino acids, carbohydrates and dietary fiber, vitamins, minerals, and adenine and hypoxanthine) [96]. Similarly, artichoke root extracts rich in inulin were used in yogurt production, showing an improvement in probiotic bacteria viability at the end of fermentation, but their use is not acceptable due to the unpleasant flavor of the product [57].

Preliminary investigation was also performed on ABPs potential applications in cheese production, as vegetable coagulant to be used as a milk-clotting agent. This property could be attributed to the presence of cardosin A and B, which lead to the production of cheese with a creamy, soft texture due to a high clotting and strong proteolytic activity on bovine k-casein [55]. Leaf extract has been identified as the most active by-products exhibiting milk-clotting and proteolytic activities [97], as recently confirmed by Esposito et al. (2016). The proteolytic activity was attributed to the aspartic protease family (common vegetable coagulant enzymes active at acidic pH and inhibited by pepstatin A) and specifically to the two aspartic acid residues [54].

ABPs were also investigated as rheological agents in *Primosale cheese* production. The flour obtained from the by-products positively increased the moisture content and reduced the cheese weight loss. This action is due to fiber component and its high water-holding capacity. In addition, the fortification with ABPs flour changed the sensory properties increasing cheese odor, aroma intensity, hardness, and gave different tactile sensations in crispness and stickiness [98].

There are other interesting applications of ABPs-based food additives regarding, mainly, bakery products, in which ABPs improve the rheological properties and increase the shelf-life of the final products [99,100]. The key factors of this action are both the high dietary fiber content, mainly insoluble fiber, and the low moisture content. In fact, this fiber component is characterized by high WHC (8.17 g/g dm) and OHC (16.17 g oil/g sample) values and, therefore, is particularly suitable for the bread preparation. A high WHC extends bread shelf-life, reducing the water loss during storage and delaying the starch degradation. The addition of 2% fiber to wheat flour improves dough properties such as its capacity to absorb water, tenacity, and stability, thanks to the high number of hydroxyl groups in the fiber skeleton that increases the interaction among fibers, gluten, and water, thus reducing the water and gluten amount required for dough development. In addition, the presence of cellulose reduces bread extensibility and softening, promoting a strong interaction among fiber and flour. The final product appears to have greater hardness and chewiness with a slightly lower specific volume and darker color [100,101]. The addition of low percentages of ABPs (<9%) is enough to change bread’s texture properties, improving its hardness, chewiness, cohesiveness, and resilience [99]. Bread can also be added with ABPs flour and this addition changes structural properties, improving hydration and stability (the shelf-life reaches several days) when compared to re-milled semolina bread, in which height, volume, and weight are reduced in few days [102].

Another application regards the addition of 4% ABPs extract to whole-wheat biscuits rich in fiber, characterized by a flavor similar to that of biscuits formulated with pea fiber and already commercially available. The main difference consists in the fact that artichoke-derived fiber makes biscuits dark colored with a hard consistence, maintaining the sensory qualities over time both during normal and accelerated storage conditions, thus increasing the product shelf-life [103]. In addition, in comparison to pea fiber products, these biscuits enriched with ABPs fiber have also a higher content of polyphenols, which is preserved even when accelerated storage conditions are applied and when biscuits are submitted to simulated in vitro digestion process. The presence of polyphenols contributes to the shelf-life increase, delaying oxidative degradation and fat rancidity. Furthermore, these biscuits retain water after cooking, having a higher moisture content than biscuits without fiber, and this makes easier the control of weight loss and excessive browning during industrial processes [104].

The antioxidant effect of ABPs rich in polyphenols was also evident in fresh egg pasta. This product, stored at 5 °C, increased its shelf-life up to more than a week [105]. Concerning fresh pasta, roots represent a valid source of inulin with high DP, which can be used as an alternative to durum wheat semolina in the pasta production. The addition of inulin preserves taste, bulkiness, adhesiveness, and hardness of the final product, decreasing product brightness and yellowness and cooking time, as fiber acts on gluten network, increasing the water penetration and the formation of hydrogen bonds and giving a lower swelling [59].

Artichoke bracts with high TPC content can be also used as wheat replacer in cake production. A 16.43% bracts was ideal to have products with high volume and softness thanks to their capacity to hold air bubbles. Conversely, the addition of a higher percentage increases the product hardness, disorganizing the starch-gluten matrix and decreasing the gas holding capacity [106].

ABPs have been also used as natural antioxidant food additives. In fact, as previously mentioned, considering their content in 5-CQA and 1,5-diCQA which possess antioxidant properties, their use in decreasing lipid and protein oxidative processes in order to improve products quality, shelf-life, and food safety were investigated. An example is the use of ABPs extracts instead of BHT in the meat industry, especially in the preservation of raw beef patties during refrigerated storage. An antioxidant activity like that obtained using BHT, which reflects an improved storage time and reduced lipid peroxidation (low primary and secondary lipid oxidation products, such as hydroperoxides, malondialdehyde) and protein oxidation (low carboxylic compounds), was registered. In addition, the inhibition of lipid and protein oxidative processes in meat products can be also related to a protective effect of extracts on heme molecule in myoglobin, thus reducing the release of iron, whose presence is responsible for meat’s red color. In fact, when oxidation occurs in meat, heme iron easily converts to non-heme iron, and this results in a brownish color and rancid aroma [107].

ABPs extracts (1000 ppm) also have antimicrobial activity, inhibiting the growth of pathogenic bacteria (*E. coli* and *L. monocytogenes*) during beef patties storage; in addition, they have a concentration-dependent action on psychrophilic and coliform bacteria [108].

ABPs were investigated as promising biosorbents for food and feed contaminants such as mycotoxins (aflatoxin B1-AFB, zearalenone-ZEA, and ochratoxin A-OTA), which are responsible for serious complications and diseases. Sorbents act by reducing mycotoxin gastrointestinal absorption and ABPs’ activity depends on pH and has an effect on toxin acidic groups; at pH 7.0, ABPs have the optimal capacity to simultaneously absorb AFB1 and ZEA, thus acting as multi-toxins binders. This effect probably depends on non-degradable fiber and on condensed tannins that are well known antinutritional agents, causing also low nutrients absorption [109].

Finally, stems were investigated as a source of peroxidase, an enzyme able to modify color and flavor of some foods and beverages (mainly beer and wine), but also an enzyme deeply studied for its chemical and biotechnological properties and used in the development of packaging materials and in diagnostic and medical devices. This new peroxidase has a strong activity at 50 °C in a pH range 6–8 using *o*-dianisidine, catechol, and guaiacol as substrates [20].

## 7. Functional Food and Food Supplements Ingredients

As above discussed, ABPs are rich in compounds with important bioactivities from antioxidant to hypoglycemic and hypolipidemic, so they represent promising functional food ingredients to enhance food healthy properties [15].

For example, stem extracts (3–9%) were added to bread and tested for their antioxidant and hypoglycemic activities after a simulated digestion process. The digested samples were active with an effect that was directly proportional to stem concentration. The antioxidant effect was due to the high duodenal bioavailability of polyphenols and cynaropicrin, while the hypoglycemic activity was related to a direct inhibition of α-glucosidase, thus potentially being applied in the modulation of glucose absorption with important diabetes prevention [53].

Similarly, the consumption of wheat flour-based chips made of bracts and omega-3-rich fish oil can have a positive impact in the prevention of cardiovascular diseases in diabetic patients. This action is due to polyphenols (mono-CQAs, di-CQAs, luteolin, apigenin, and their glycosylated derivatives) and PUFAs, as demonstrated using diabetic mice as animal model. A reduction in blood glucose level, TGs, total cholesterol, and LDL, and an increase in HDL was registered [73].

Artichokes leaf extracts added to gluten-free bread can be considered a functional food ingredient for subjects with celiac disease. The extracts increase bread antioxidant and anti-inflammatory properties, protecting it from oxidative stress and reducing the expression of pro-inflammatory mediators (cytokines and interleukins) [110].

Finally, the antioxidant capacity of ABPs has been also investigated for the production of chicken soups, fresh pasta, and spreadable cheese [46,111,112]. An interesting application in the production of functional fresh pasta is the use of ABPs inulin as ingredient; the obtained food product possesses prebiotic properties, with a protective effect against pathogenic bacteria (*E. coli*) and colorectal disorders. This enriched pasta also exhibited important effects in the modulation of glucose and lipids [59].

ABPs extracts also represent a promising source of active ingredients for food supplements preparation. They exhibited hypolipidemic and hypoglycemic properties as above mentioned, also further confirmed by clinical studies. A hypolipidemic activity was confirmed in a study conducted on patients with chronic kidney disease treated with artichoke leaf extract for six weeks. It was demonstrated that there was a direct effect on the reduction of total cholesterol and LDL, which also correlated to a decreased appetite, without the involvement of TGs and HDL levels [113]. Similarly, a reduction of lipids (particularly TGs and total cholesterol) and liver enzymes (alanine and aspartate aminotransferases) (ALT) levels were evident in nonalcoholic steatohepatitis patients [114].

## 8. Future Prospectives of Artichoke By-Products Use

### 8.1. Health and Nutrition Claims

Regarding *Cynara scolymus* L. and its parts admitted in the preparation of food supplements, the Belfrit list (last update 2018) drawn up by Belgium, France, and Italy and notified to the EC, includes head (edible part) and leaf, without any specification about leaf types. Many effects have been declared for leaf, from digestive and depurative to antioxidant with specific activities on liver function and lipid metabolism [115].

As regards EU claims, only one health claim about the correlation between the globe artichoke consumption and the increase in renal water elimination has been proposed, resulting non-authorized by EFSA in 2010. The other two non-authorized health claims (2011) do not concern artichoke as “nutrient substance food or food category”, but the reduction of gastro-intestinal discomfort and the potential decrease of pathogenic gastro-intestinal microorganisms. Therefore, the main focus is the action on the proliferation of probiotic Lactobacillus paracasei LMG P-22043 added to artichoke [116,117,118].

The potential effect of artichoke and ABPs on kidney could obtain a real health claim in the future [116]. In fact, as commented on above, ABPs extracts have a positive role in clinical studies enrolling patients with chronic kidney disease associated with dyslipidemia. However, this effect is due to a hypolipidemic activity with no effect on renal water elimination [113]. The real possibility to take advantage of ABPs extract hypolipidemic action [71,72,73] has been also confirmed by a recent authorized health claim (2023) for a combination of artichoke leaf dry extract standardized in caffeoylquinic acids with monacolin K (from red yeast rice), sugar-cane derived policosanols, oligomeric proanthocyanidin (OPC) from French maritime pine bark, garlic dry extract standardized in allicin, d-α-tocopheryl hydrogen succinate, riboflavin, and inositol hexanicotinate [119,120]. This claim concerns the “reduction of blood LDL-cholesterol concentrations” and although it has been authorized for a complex mixture, including other compounds with well-known effects on lipid metabolism; it could open the possibility of future request for ABPs extract claims on “fat metabolism” [116].

In addition, it is important to mention that a high number of food supplements containing artichoke extract as ingredient for its health effects are on the market without any health claim; therefore, ABPs components could be promising ingredient (after approving them as novel foods, if necessary) without the mandatory request for a health claim. Finally, a possible interesting nutritional claim could be “source of fiber”, considering the high content in inulin, arabinans, arabinogalactans, and xyloglucans (with action on intestinal lipid absorption) (See Section 4.1).

The possibility to obtain the approval for an ABPs health claim could be real if we consider that the Committee on Herbal Medicinal Products (HMPC) of the European Medicines Agency (EMA) declaration on artichoke leaf preparations. In fact, the dried basal leaves of *Cynara scolymus* L., containing a minimum 0.8% of 5-CQA, are present in different herbal preparations and pharmaceutical forms, often combined with other products. These products are actually present on the market of some European countries and their suggested use is mainly for functional dyspepsia and digestive problems, and sometimes for their lipid lowering effect [121].

### 8.2. Novel Foods

Novel foods are defined as foods or food components/ingredients that have not been consumed to a significant degree by humans in the EU before the first regulation has been established, i.e., 15 May 1997. They can be derived by new process and/or production technology or consist of food or herbal extract traditionally consumed outside EU [122].

Nowadays, the novel foods list includes also seeds and seed oils characterized by a high fatty acids content, leaves, and root extracts that can be used for the production of bakery products, fruit juice, and fruit/vegetable blend beverages or food supplements, mainly for the high content in proteins and fiber of leaves ad roots [123,124].

ABPs could represent new plant-based foods, meeting the growing market request for plant-based products. In fact, globe artichoke stem, bract, and residual leaf polyphenols and fiber can be considered the most promising ingredients present in such extracts and good candidates for the inclusion in the novel foods category. If approved as novel foods, ABPs polyphenols could be introduced in the market once added to bakery products, as bread and chips, with antioxidant and hypoglycemic effects, as previously mentioned [53,73]. In addition, also ABPs inulin added to pasta products exhibited a positive activity in the control of glucose in addition to its prebiotic effect [59].

## 9. Animal Feed

Over the years, to solve the problem of increased needs and cost of intensive livestock farms and feed production, different animal feed sources have been investigated. By-products attracted the attention because considered potential useful alternatives with the aim also to reduce waste disposal management. In addition, the presence of bioactives in by-products can improve the nutritional value of feed and by consequence animal health. ABPs ensilage represents an important procedure to obtain available and stable feeds storable for long time independent of artichoke’s season [21,29].

The process of ensiling ABPs in big commercial-sized silos can produce stable extracts from a microbiological and nutritional point of view for more than six months. A stable and low pH, a high lactic acid content, anaerobic atmosphere, and sugar absence ensure a reduction in the growth of dangerous microorganisms. All these conditions also improve the quality of the products, decrease the risk of nutrients lost (mainly proteins) and increase the content of active molecules, such as polyphenols. Lactic acid bacteria produce β-glucosidase enzyme which is involved in phenols release during the ensiled fermentation. In addition, the obtained animal feed has low levels of ethanol, volatile fatty acids (propionic, butyric, and isobutyric acids), and contamination by enterobacteria, clostridia, and yeasts, and, therefore, it is safer [29]. So, ABPs are suitable for ensilage production, not only for the nutritional and microbiological advantages above mentioned, but also for the improvement in organoleptic properties, such as palatability and smell of milk and derivatives (cheese and yogurt) [125].

For example, the incorporation of ensiling outer bracts in different amount (0–25%) in the diet of dairy goat affected the content of proteins and fiber, decreasing them with a small change in milk color, and no formation of off-flavors. No changes in acidification ability, lactose and lactic acid bacteria quantities, coagulating properties were registered [126]. Regarding lipids content, the ensiled bract had high content in oleic acid, PUFA, and conjugated linoleic acid, with interesting anti-atherogenic properties and anti-obesity activity [29]. The ensiling bracts (40%) added to goat’s milk confirmed no changes in milk composition and production but had an effect on lipid and mineral (higher concentrations of calcium, zinc, manganese, and copper) content [30,127]. Table 3 contains the different food applications of ABPs (bracts, stems, residual leaves, and roots).

## 10. Conclusions

Globe artichoke by-products, particularly bracts and stems, are rich in components with functional and/or biological properties exploitable in different food applications, to produce functional foods, food supplements, and animal feed. The health implications of these products are evident, thanks to their composition of bioactive molecules that possess a high number of properties useful in the prevention of many important chronic diseases. The promising re-use of such high amounts of artichokes’ non-edible parts represents an important example of circular economy. In addition, the possibility to apply green methodologies, also scalable at industrial level, allowed for obtaining rapid and efficient extractions, maximizing yields and time in complete sustainable recovery processes to produce active ingredients. Bracts and stems represent the main ABPs that are potentially re-usable, being rich in bioactive compounds exerting biological and/or functional properties that are useful in different healthy applications. The most important application can be considered as their use as additives or bioactive ingredients in food industries to produce functional foods, foodstuffs, and food supplements. In addition, some extracted compounds can be also investigated for non-food applications in pharmaceutical and cosmetic industries due to their healthy and technological properties. Finally, also non-healthy applications should be considered, in particular for insoluble fiber, which is good source for bioenergy, polymers, and sorbents production.

## Figures and Tables

**Table 1:** Biological properties of artichoke by-products.

Artichoke By-Product	Biological Property	Reference
	Anti-inflammatory	[45]
	Antioxidant	[16]
	Prebiotic	[21]
Bract	Hypolipidemic	[73]
	Antiglycative	[44]
	Antimicrobial	[14]
	Antiproliferative	[82]
	Antioxidant	[81]
	Anti-inflammatory	[77]
Stem	Prebiotic	[21]
	Antiglycative	[44]
	Antimicrobial	[12]
	Antiproliferative	[82]
	Antioxidant	[16]
Residual	Anti-inflammatory	[52]
Leaf	Hypoglycemic	[74]

**Table 2:** Functional properties of artichoke by-products.

Artichoke By-Product	Rheological Property	Color Parameters	Reference
Bract	Emulsifier property	a*, b*	[34,89,90]
	Gelling property		
	WHC ^a^		
	OHC ^b^		
Stem	Emulsifier property	a*, b*, L*	[34,89,90]
	Gelling property		
	WHC ^a^		
	OHC ^b^		
Root	Gelling property		[58]

^a^ Water-holding capacity. ^b^ Oil-holding capacity.

**Table 3:** Food application of artichoke by-products.

Artichoke By-Product	Food Additive	Functional Food	Food Supplement	Animal Feed	Reference
Bract	Rheological additive	Wheat-flour-based chips	-	Ensilage	[29,73,102,104]
	Wheat replacer	Chicken soup	-	-	[106,111]
	Preservative		-	-	[102,105,107]
	Biosorbent		-	-	[109]
Stem	Reological additive	Bread	-	Ensilage	[29,53,100]
	Preservative	Spreadable cheese	-	-	[100,105,112]
	Biosorbent	-	-	-	[109]
	Modifier, stabilizer, off-flavor sequestrant	-	-	-	[20]
Residual leaf	Thickener	Gluten-free bread	Hypolipidemic ingredient	-	[96,110,113]
	Stabilizer	-	-	-	[96]
	Milk-clotting agent	-	-	-	[97]
	biosorbent	-	-	-	[109]
Root	Rheological additive	Fresh pasta	-	-	[59]
	Stabilizer	-	-	-	[57]

## Data Availability

No new data were created or analyzed in this study. Data sharing is not applicable to this article.
